# Reference Percentiles for Bioelectrical Phase Angle in Athletes

**DOI:** 10.3390/biology11020264

**Published:** 2022-02-08

**Authors:** Francesco Campa, Diana Maria Thomas, Krista Watts, Nicholas Clark, Daniel Baller, Thomas Morin, Stefania Toselli, Josely Correa Koury, Giovanni Melchiorri, Angela Andreoli, Gabriele Mascherini, Cristian Petri, Luis Bettencourt Sardinha, Analiza Monica Silva

**Affiliations:** 1 Department for Life Quality Studies, University of Bologna, 47921 Rimini, Italy; 2 Department of Mathematical Sciences, United States Military Academy, West Point, NY 10996, USA; diana.thomas@westpoint.edu (D.M.T.); krista.watts@westpoint.edu (K.W.); nicholas.clark@westpoint.edu (N.C.); daniel.baller@westpoint.edu (D.B.); thomas12vm@gmail.com (T.M.); 3 Departments of Biomedical and Neuromotor Sciences, University of Bologna, 40121 Bologna, Italy; stefania.toselli@unibo.it; 4 Department of Basic and Experimental Nutrition, Nutrition Institute, State University of Rio de Janeiro, Rio de Janeiro 20550-900, Brazil; jckoury@gmail.com; 5 Department of Systems Medicine, University of Tor Vergata, 00175 Rome, Italy; gmelchiorri@libero.it (G.M.); angela.andreoli@uniroma2.it (A.A.); 6 Department of Experimental and Clinical Medicine, University of Florence, 50134 Florence, Italy; gabriele.mascherini@unifi.it; 7 Department of Sports and Computer Science, Section of Physical Education and Sports, Universidad Pablo de Olavide, 41013 Seville, Spain; cpet2@alu.upo.es; 8 Exercise and Health Laboratory, CIPER, Faculdade de Motricidade Humana, Universidade de Lisboa, 1499-002 Cruz Quebrada, Portugal; lbsardinha55@gmail.com (L.B.S.); analiza@fmh.ulisboa.pt (A.M.S.)

**Keywords:** BIA, body composition, fat-free mass, endurance, sports performance, team sports

## Abstract

**Simple Summary:**

The bioelectrical phase angle is a raw parameter that can be utilized as an indicator of performance, muscle quantity and hydration status of cells. However, sex- and sport-specific phase angle reference percentiles are lacking for the athletic population. For the first time, this study provides 5th, 15th, 50th, 85th, and 95th reference percentiles for phase angle in male and female athletes practicing different sports. These reference values can be used to track body composition and performance related-outcomes in sports practice, while leveraging the portability of bioelectric impedance analysis.

**Abstract:**

The present study aimed to develop reference values for bioelectrical phase angle in male and female athletes from different sports. Overall, 2224 subjects participated in this study [1658 males (age 26.2 ± 8.9 y) and 566 females (age 26.9 ± 6.6 y)]. Participants were categorized by their sport discipline and sorted into three different sport modalities: endurance, velocity/power, and team sports. Phase angle was directly measured using a foot-to-hand bioimpedance technology at a 50 kHz frequency during the in-season period. Reference percentiles (5th, 15th, 50th, 85th, and 95th) were calculated and stratified by sex, sport discipline and modality using an empirical Bayesian analysis. This method allows for the sharing of information between different groups, creating reference percentiles, even for sports disciplines with few observations. Phase angle differed (men: *p* < 0.001; women: *p* = 0.003) among the three sport modalities, where endurance athletes showed a lower value than the other groups (men: vs. velocity/power: *p* = 0.010, 95% CI = −0.43 to −0.04; vs. team sports: *p* < 0.001, 95% CI = −0.48 to −0.02; women: vs. velocity/power: *p* = 0.002, 95% CI = −0.59 to −0.10; vs. team sports: *p* = 0.015, 95% CI = −0.52 to −0.04). Male athletes showed a higher phase angle than female athletes within each sport modality (endurance: *p* < 0.01, 95% CI = 0.63 to 1.14; velocity/power: *p* < 0.01, 95% CI = 0.68 to 1.07; team sports: *p* < 0.01, 95% CI = 0.98 to 1.23). We derived phase angle reference percentiles for endurance, velocity/power, and team sports athletes. Additionally, we calculated sex-specific references for a total of 22 and 19 sport disciplines for male and female athletes, respectively. This study provides sex- and sport-specific percentiles for phase angle that can track body composition and performance-related parameters in athletes.

## 1. Introduction

Body composition assessment is an important practice in sports management, given the numerous implications on health and physical performance [1]. Several compartments of body composition are predictors of health and sports performance outcomes [1]. For example, a higher percent body fat is negatively related with the quality of movement and physical performance, in particular sports that require sprinting or jumping [2]. Additionally, muscle mass contributes to the production of strength and power [3] and total body water influences neuromuscular, as well as cognitive functions [4,5,6]. The most accurate body composition assessment evaluations depend on specialized laboratory based methods, such as magnetic resonance imaging, isotope dilution techniques, dual-energy X-ray absorptiometry, and air plethysmography [7]. These laboratory-based body composition assessments quantify tissue (e.g., lean soft and skeletal muscle tissues) and molecular-level (e.g., total body water) body composition elements. However, the amount of specialized training required, high cost, and time-burden to athletes being measured make such methods unfeasible for routine application by coaches, trainers or other sports managers [7]. 

Due to the portability and ease of application, the use of the bioelectrical impedance analysis (BIA) has been increasing in sports practice especially because current studies have revealed that when following standardized protocols for BIA assessment, body composition measurements are comparable to more sophisticated clinical methods [7,8,9,10]. In addition, BIA yields additional raw bioelectrical parameters that can be independently used to qualitatively track body composition [7,8]. Particularly, bioelectrical resistance and reactance, the two components of impedance, are associated with body fluid content and cell density, respectively [11]. The bioelectrical phase angle, which is the arctangent of reactance divided by resistance, has been identified as a biomarker of muscle quantity and predicts the intracellular-to-extracellular water ratio in athletes [7,8]. In particular, high phase angle values are associated with subjects who have high muscle mass and a higher content of intracellular than extracellular fluids [12,13,14]. In the context of sports, changes in phase angle are associated with physical adaptations obtainable after training or nutritional interventions [8]. Furthermore, recent findings have highlighted the phase angle’s positive association with sport-specific muscle strength and power test outcomes [15,16,17]. 

The relationships between phase angle and body composition have led to a call for methods that leverage phase angle data to track athletic performance and health over a season [18]. To date, reference phase angle values are available for the general and elderly population [19,20,21,22,23]. However, to the best of our knowledge, reference phase angle percentiles for athletes do not exist. To fill this gap, the present study aimed to develop sex- and sport-specific phase angle reference percentiles for athletes. 

## 2. Materials and Methods

### 2.1. Participants and Study Design

This cross-sectional study involved 2224 national athletes assessed in the in-season period and including 1658 males (age 26.2 ± 8.9 y) and 566 females (age 26.9 ± 6.6 y) of 22 and 19 different sport disciplines, respectively. The following inclusion criteria were used: (i) aged 16 y or older; and (ii) not injured or ill at the moment of the test. Similar to previous studies [1,24], the participants were sorted into three groups of sport modalities: endurance (cycling, marathon, pentathlon, sailing, ski, rowing, and triathlon), velocity/power (athletics including jumping, throwing, short-distance running; badminton; boxing; judo; karate; kickboxing; rhythmic gymnastics; swimming including short-distance swimming; and tennis), and team sports (basketball, field hockey, handball, rugby, soccer, volleyball, and water polo). Since they cannot be considered as athletes [18], soccer referees and CrossFit exercisers were not included in any of the three sports modalities. All subjects were informed of the study design before giving written informed consent to participate. As sample sizes within some sports were low, percentiles were estimated using a parametric and empirical Bayesian framework that allowed for information sharing between sports. All procedures were approved by the bioethics committee of the University of Bologna and were conducted in accordance with the declaration of Helsinki for human studies (Ethical Approval Code: 25027; dated 13 March 2017).

### 2.2. Procedures

All measurements were performed in resting conditions (9.00 AM) following standard procedures suggested for athletes [7,25]. Height was measured to the nearest 0.1 cm using a stadiometer. Body weight was determined to the nearest 0.1 kg, using a mechanical scale (Seca 711, Seca, Hamburg, Deutschland). Body mass index (BMI) was calculated as total body mass (kilograms) divided by height (meters) squared. BIA was performed according with standardized procedures specific for athletes [9]. The bioelectrical phase angle was directly measured with BIA (BIA 101, Akern, Florence, Italy), which applies an alternating current of 800 μA at a single frequency of 50 kHz. Fat-free mass (FFM) (kilograms) was estimated using a specific equation for athletes [26] and FFM index (FFMI) calculates as FFM divided by height (meters) squared.

### 2.3. Statistical Analysis

**Figure 1:**
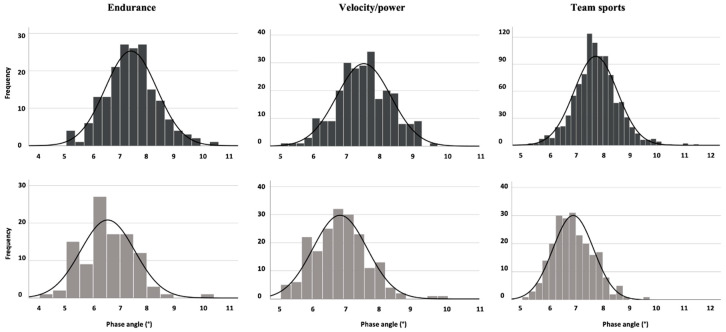
Phase angle distribution by sport modality and sex, with men and women in upper and lower panels, respectively.

Statistical analyses were performed using the R software (version 4.1.0). Analyses were performed to complete two tasks: (i) estimate the reference percentiles for phase angle, stratified by sex and sport; (ii) test whether the mean for each outcome differed by sex and sport modalities. To create reference percentiles, an empirical Bayesian analysis was performed [27]. Empirical Bayesian analysis allows the sharing of information across sports to augment our inference whenever we had at least two athletes’ values. This allows estimates for sports where there are only a few participants. In order to conduct the analysis, both a sampling distribution as well as a prior distribution for the parameters of the sampling distribution must be specified. Within a given sex and sport, the athletes’ outcome values were assumed to follow a normal (Gaussian) distribution that could be characterized through its mean and precision (inverse variance). This assumption is supported based on the sample distributions, as showed in Figure 1. If the mean and precision were known, all quantiles followed immediately from the normal assumption. The sport-specific means and variances were modelled as arising from a normal-gamma distribution, which serves as the prior and forms a conjugate family with our observational model. The hyperparameters of the prior were informed empirically through maximum-likelihood by using all athletes’ data for this outcome, restricted by sex. Once this was carried out, joint posterior distributions for the mean and precision were generated for every sport, giving rise to point estimates and 95% joint confidence regions for the mean and precision, which in turn were used to calculate simultaneous 95% confidence intervals for the reference percentiles of interest. Percentiles (5th, 15th, 50th, 85th, and 95th) were presented for all sports/sex subgroups. Sex and sport modality comparisons for phase angle and FFMI were performed with unpaired t-tests or analysis of variance with Bonferroni post-hoc. Statistical significance was predetermined as *p* < 0.05. Cohen’s d effect size (ES) with 95% confidence interval (CI) was reported for significant between-sex comparisons and interpreted according to the following Hopkins’ recommendations: 0–0.19: trivial; 0.20–0.59: small; 0.60–1.19: moderate; 1.20–1.99: large; ≥2.00: very large [28].

## 3. Results

Table 1 presents the data summary for each sport discipline. 

Table 2 shows the general characteristics for the athletes grouped by sex and sport modality. Phase angle differed (men: *p* < 0.001,; women: *p* = 0.003,) among the three sport modalities, where endurance athletes showed a lower value than the other groups (men: vs. velocity/power: *p* = 0.010; vs. team sports: *p* < 0.001; women: vs. velocity/power: *p* = 0.002; vs. team sports: *p* = 0.015). Male athletes showed a higher phase angle than female athletes within each sport modality (endurance: *p* < 0.01, ES = 0.88, 95% CI = 0.63 to 1.14; velocity/power: *p* < 0.01, ES = 0.88, 95% CI = 0.68 to 1.07; team sports: *p* < 0.01, ES = 1.12, 95% CI = 0.98 to 1.23).

Significant differences were found for FFMI among sport modalities (men: *p* = 0.002; women: *p* = 0.025). Endurance showed a mean FFMI value lower than velocity/power (men: *p* = 0.033; women: *p* = 0.048) and team sports athletes (men: *p* = 0.001; women: *p* = 0.038). Male athletes showed a higher FFMI than female athletes within each sport modality (endurance: *p* < 0.01, ES = 2.21, 95% CI = 1.82 to 2.41; velocity/power: *p* < 0.01, ES = 1.80, 95% CI = 1.55 to 2.04; team sports: *p* < 0.01, ES = 1.59, 95% CI = 1.44 to 1.74). 

The sport and sex derived reference percentiles 5th, 15th, 50th, 85th, and 95th for phase angle values by sport disciplines and modalities are reported in Table 3 and Table 4 for men and women, respectively. 

## 4. Discussion

The present study aimed to derive phase angle reference percentiles for athletes of both sexes and involved in different sports. To the best of our knowledge, this is the first study that provides normative values for phase angle in an athletic population, which until now were only available for the general and elderly population [19,20]. 

The results of this study showed that regardless of sports modality, male athletes have a higher phase angle than their female counterparts. These results are in congruence with previous investigations conducted in the general population and in former athletes [15,19]. The biological mechanism for this observation may be due to the positive correlation of phase angle with muscle mass and the intra-to-extracellular water ratio [12,29], two parameters generally higher in male subjects [30]. Since phase angles differ within a sport by player position and role [31], it becomes difficult to compare values across sports disciplines without carefully considering these specific-factors. Differences in athletes’ phase angle among different game roles, can be due to the requirements of the specific position that influences specific body composition features, especially in team sports [32]. To mitigate this challenge, comparisons between sports are often made classifying the athletes by type of sport activity, such as endurance, velocity/power, and team sports [1,24]. Between sport classifications, athletes of both sexes involved in endurance sports retained a lower phase angle compared to those engaged in velocity/power and team sports. The difference in phase angles across these sports modalities is possibly due to the lower muscle mass required for endurance compared to sports where strength and power are necessary [1]. However, the magnitude of the differences was small and further speculation could be misleading in this context. Similarly, endurance athletes of both sexes exhibited lower FFMI than other categories. This finding is probably due to more FFM present in velocity/power or team sports athletes for who the sport demand is typically anaerobic. In fact, some FFM compartments such as muscle tissue are particularly important for the glycolytic mechanisms of energy production [33]. 

This study reported the 5th, 15th, 50th, 85th, and 95th reference percentiles for athletes’ phase angles by sex and sport. To aid application of the newly developed percentiles, we provide an example for a male endurance athlete with a phase angle of 6.8°. Table 3 shows that the estimate for the 5th percentile is 6.2° (95% CI: 6.1 to 6.3°), the estimate for the 15th percentile is 6.7° (95% CI: 6.7 to 6.8°), and the estimate for the 50th percentile is 7.6° (95% CI: 7.6 to 7.7°). Thus, the phase angle of this hypothetical male endurance athlete falls in the bottom half of the distribution but above the lowest 5% of that distribution, with an estimated value close to the 15th percentile for his sport category. Since phase angle usually decreases after a preliminary time period [34,35] due to the higher initial training load, this endurance athlete may undergo a decrease in phase angle beyond the 15th percentile over the season. In this study, BIA was performed during the main time of competition of the in-season period. An ideal measurement during the in-season period should stay above the 50th percentile for an athlete who is healthy and in optimal physical condition to tackle this phase of the season. Given that phase angle primarily reflects cellular health and hydration and muscle mass and its quality, nutritional support strategies should be considered, especially in athletes with low phase angle values [36,37,38]. 

The problem of recognizing exercisers who, having body composition characteristics similar to those of athletes, would benefit from specific prediction formulas for athletes even if they do not fall into this category, has recently been discussed [18]. In this regard, evaluating phase angle as a pre-screening biomarker may help to identify subjects with bioelectrical and body composition characteristics similar to the athletic population rather than to those of the general population. For example, while Mattiello et al. [19] identified a phase angle at the 50th percentile of 7.0° for an adult male belonging to the general population, in this study the 50th percentile of a male athlete is around 7.7°. The same scenario occurs for female athletes where higher mean values than the general healthy population have previously been identified [19]. However, previous studies have shown how exercisers can still have phase angle values similar to those of an athlete [4,39]. Therefore, the phase angle reference values provided in this study can help practitioners understand when a subject from the general population may still have body composition characteristics similar to those of an athlete. Along these lines, future research is needed to develop phase angle cut-off values useful to identify when a BIA-based athlete-specific equation should be used to estimate body composition even in subjects belonging to the general population.

A strength of this study is the database consisting of a large number of athletes measured during the in-season phase. This is important because body composition and as a result phase angles change across the competitive season phases [34,35]. Some limitations are present in this study. First, we have not been able to provide reference values for young athletes, in which an increase in the phase angle is mainly dictated by the state of maturity that acts by modifying the characteristics of body composition [7,40]. In this regard, future research should fill this gap by including youth groups and measuring phase angle and maturity offset. Second, our outcomes were obtained from the use of a foot-to-hand technology (BIA 101, Akern, Florence, Italy), and a 50 kHz sampling frequency. Therefore, given the lack of agreement between BIA technologies and sampling frequencies [41,42,43], the current findings cannot be extended to different technologies (e.g., BIA in standing position) and sampling frequencies. Lastly, although the use of empirical Bayesian framework allowed us to ‘share’ information across sports with lower number of participants, measures of sports disciplines with high sample sizes may have an outsized influence on the estimates of the smaller sports. However, we considered three large group of sport modalities in order to improve the quality of the athletes’ classification when assessing phase angle in research as well as in the field context. 

## 5. Conclusions

This study derived phase angle normative values for male and female athletes of different sports. Considering the usefulness of phase angle as a marker of a healthier body composition profile and performance, coaches and practitioners will benefit from using these sex and sports specific reference percentiles in assessing and tracking athletes over a season. These findings will also help to establish directions for future research in sports practice.

## Figures and Tables

**Table 1:** Participants’ data summary.

Men		Women	
Sport Discipline	N (% of Total)	Sport Discipline	N (% of Total)
Overall	1658 (100%)	Overall	566 (100%)
Athletics	47 (2.8%)	Athletics	19 (3.4%)
Badminton	7 (0.4%)	Badminton	4 (0.7%)
Basket	85 (5.1%)	Basket	55 (9.7%)
CrossFit	61 (3.7%)	Boxing	9 (1.6%)
Cyclists	15 (0.9%)	CrossFit	41 (7.2%)
Field Hockey	12 (0.7%)	Gymnastics	25 (4.4%)
Handball	40 (2.4%)	Handball	4 (0.7%)
Judo	53 (3.2%)	Judo	23 (4.1%)
Karate	28 (1.7%)	Karate	5 (0.9%)
Kick-boxing	48 (2.9%)	Kick-boxing	20 (3.5%)
Marathon	80 (4.8%)	Marathon	53 (9.4%)
Pentathlon	32 (1.9%)	Pentathlon	20 (3.5%)
Rugby	147 (8.9%)	Rowing	13 (2.3%)
Sailing	2 (0.1%)	Soccer	55 (9.7%)
Ski	4 (0.2%)	Swimming	46 (8.1%)
Soccer	490 (29.6%)	Tennis	42 (7.4%)
Soccer referees	149 (9.0%)	Triathlon	20 (3.5%)
Swimming	49 (3.0%)	Volleyball	94 (16.6%)
Tennis	25 (1.5%)	Waterpolo	18 (3.2%)
Triathlon	51 (3.1%)		
Volleyball	174 (10.5%)		
Waterpolo	59 (3.6%)		

**Table 2:** Body composition characteristics of the athletes according to sport modality.

	N	Age (y)	Weight (kg)	Height (cm)	BMI (kg/m^2^)	FFM (kg)	FFMI (kg/m^2^)	Phase Angle (°)
Men								
Endurance	182	27.9 (11.4)	70.6 (8.9)	175.5 (6.4)	22.9 (2.5)	61.7 (6.5)	20.0 (1.7)	7.4 (0.9)
Velocity/power	257	25.6 (9.9)	73.4 (10.6)	177.2 (7.8)	23.2 (2.5)	65.3 (8.5)	20.6 (2.2)	7.6 (0.9)
Team sports	1007	23.6 (6.1)	80.9 (13.4)	184.1 (9.6)	23.9 (3.8)	70.4 (10.6)	20.7 (2.6)	7.7 (0.8)
Women								
Endurance	106	33.6 (13.1)	60.6 (7.9)	166.3 (8.1)	21.9 (2.6)	45.4 (6.0)	16.2 (1.4)	6.5 (1.0)
Velocity/power	213	27.8 (10.2)	61.5 (9.2)	166.9 (6.8)	21.9 (2.2)	47.5 (7.5)	17.2 (1.7)	6.9 (0.9)
Team sports	227	28.0 (7.0)	67.2 (9.2)	174.1 (10.0)	22.1 (2.2)	51.5 (7.2)	17.0 (1.6)	6.8 (0.8)

Note: Data are reported as mean (standard deviation); BMI: body mass index; FFM: fat-free mass; FFMI: fat-free mass index.

**Table 3:** Phase Angle reference percentiles for men.

Sport Discipline	5th (95% CI)	15th (95% CI)	50th (95% CI)	85th (95% CI)	95th (95% CI)
Athletics	6.3 (6.2, 6.3)	6.8 (6.7, 6.8)	7.7 (7.6, 7.7)	8.6 (8.5, 8.6)	9.1 (9.0, 9.1)
Badminton	6.2 (6.1, 6.3)	6.8 (6.7, 6.9)	7.6 (7.6, 7.7)	8.5 (8.4, 8.6)	9.0 (8.9, 9.1)
Basketball	6.3 (6.2, 6.3)	6.8 (6.7, 6.8)	7.6 (7.6, 7.7)	8.5 (8.4, 8.6)	9.0 (8.9, 9.1)
CrossFit	6.3 (6.2, 6.3)	6.8 (6.7, 6.8)	7.6 (7.6, 7.7)	8.5 (8.4, 8.6)	9.0 (8.9, 9.1)
Cyclists	6.3 (6.2, 6.4)	6.8 (6.7, 6.9)	7.6 (7.6, 7.7)	8.5 (8.4, 8.6)	9.0 (8.9, 9.1)
Field hockey	6.3 (6.2, 6.4)	6.8 (6.7, 6.9)	7.6 (7.6, 7.7)	8.5 (8.4, 8.6)	9.0 (8.9, 9.1)
Handball	6.3 (6.2, 6.4)	6.8 (6.7, 6.9)	7.7 (7.6, 7.7)	8.5 (8.5, 8.6)	9.0 (8.9, 9.1)
Judo	6.3 (6.2, 6.4)	6.8 (6.7, 6.9	7.6 (7.6, 7.7)	8.5 (8.4, 8.6)	9.0 (8.9, 9.1)
Karate	6.3 (6.2, 6.4)	6.8 (6.7, 6.9)	7.6 (7.6, 7.7)	8.5 (8.4, 8.6)	9.0 (8.9, 9.1)
Kick-boxing	6.3 (6.2, 6.3)	6.8 (6.7, 6.8)	7.6 (7.6, 7.7)	8.5 (8.4, 8.5)	9.0 (8.9, 9.1)
Marathon	6.3 (6.2, 6.3)	6.8 (6.7, 6.8)	7.6 (7.6, 7.7)	8.5 (8.4, 8.5)	9.0 (8.9, 9.1)
Pentathlon	6.3 (6.2, 6.4)	6.8 (6.7, 6.8)	7.6 (7.6, 7.7)	8.5 (8.4, 8.6)	9.0 (8.9, 9.1)
Rugby	6.3 (6.2, 6.4)	6.8 (6.7, 6.9)	7.6 (7.6, 7.7)	8.5 (8.4, 8.6)	9.0 (8.9, 9.1)
Sailing	6.3 (6.2, 6.4)	6.8 (6.7, 6.9)	7.6 (7.6, 7.7)	8.5 (8.4, 8.6)	9.0 (8.9, 9.1)
Ski	6.3 (6.2, 6.4)	6.7 (6.7, 6.9)	7.7 (7.6, 7.7)	8.5 (8.4, 8.6)	9.0 (8.9, 9.1)
Soccer	6.3 (6.2, 6.4)	6.8 (6.7, 6.9)	7.6 (7.6, 7.7)	8.5 (8.4, 8.6)	9.0 (8.9, 9.1)
Soccer referees	6.3 (6.2, 6.3)	6.8 (6.8, 6.8)	7.6 (7.6, 7.7)	8.5 (8.5, 8.6)	9.0 (8.9,9.1)
Swimming	6.3 (6.2, 6.4)	6.8 (6.7, 6.9)	7.6 (7.6, 7.7)	8.5 (8.4, 8.6)	9.0 (9.0, 8.9)
Tennis	6.3 (6.2, 6.4)	6.8 (6.7, 6.9)	7.6 (7.6, 7.7)	8.5 (8.4, 8.6)	9.0 (8.9, 9.1)
Triathlon	6.3 (6.2, 6.4)	6.8 (6.7, 6.9)	7.7 (7.6, 7.7)	8.5 (8.4, 8.6)	9.0 (8.9, 9.1)
Volleyball	6.3 (6.2, 6.4)	6.8 (6.7, 6.9)	7.6 (7.6, 7.7)	8.5 (8.4, 8.5)	8.9 (8.9, 9.0)
Waterpolo	6.3 (6.2, 6.4)	6.8 (6.7, 6.9)	7.6 (7.6, 7.7)	8.5 (8.4, 8.6)	9.0 (8.9, 9.1)
Sport modality					
Endurance	6.2 (6.1, 6.3)	6.7 (6.7, 6.8)	7.6 (7.6, 7.7)	8.5 (8.5, 8.6)	9.1 (9.0, 9.1)
Velocity/power	6.3 (6.2, 6.4)	6.8 (6.8, 6.9)	7.7 (7.7, 7.8)	8.6 (8.5, 8.7)	9.1 (9.0, 9.2)
Team sports	6.3 (6.2, 6.3)	6.8 (6.7, 6.8)	7.7 (7.6, 7.8)	8.5 (8.5, 8.6)	9.1 (9.0, 9.1)

**Table 4:** Phase Angle reference percentiles for women.

Sport Discipline	5th (95% CI)	15th (95% CI)	50th (95% CI)	85th (95% CI)	95th (95% CI)
Athletics	5.4 (5.3, 5.5)	5.9 (5.8, 6.0)	6.8 (6.7, 6.8)	7.7 (7.6, 7.8)	8.2 (8.1, 8.3)
Badminton	5.4 (5.3, 5.5)	5.9 (5.8, 6.0)	6.8 (6.8, 6.9)	7.7 (7.6, 7.8)	8.2 (8.1, 8.4)
Basketball	5.4 (5.3, 5.5)	5.9 (5.9, 6.0)	6.9 (6.8, 6.9)	7.7 (7.6, 7.8)	8.2 (8.1, 8.3)
Boxing	5.4 (5.3, 5.5)	5.9 (5.9, 6.0)	6.9 (6.8, 7.0)	7.7 (7.6, 7.9)	8.3 (8.1, 8.4)
CrossFit	5.3 (5.2, 5.4)	5.9 (5.8, 6.0)	6.8 (6.8, 6.9)	7.7 (7.6, 7.8)	8.2 (8.1, 8.3)
Gymnastics	5.4 (5.3, 5.5)	5.9 (5.9, 6.0)	6.8 (6.8, 6.9)	7.8 (7.7, 7.9)	8.3 (8.1, 8.4)
Handball	5.4 (5.3, 5.5)	5.9 (5.8, 5.9)	6.8 (6.7, 6.9)	7.7 (7.6, 7.8)	8.2 (8.1, 8.4)
Judo	5.4 (5.3, 5.5)	5.9 (5.8, 5.9)	6.8 (6.7, 6.9)	7.8 (7.7, 7.9)	8.3 (8.1, 8.4)
Karate	5.4 (5.4, 5.5)	5.9 (5.9, 6.0)	6.8 (6.8, 6.9)	7.7 (7.6, 7.8)	8.2 (8.1, 8.4)
Kick-boxing	5.4 (5.4, 5.5)	5.9 (5.9, 6.0)	6.8 (6.8, 6.9)	7.7 (7.6, 7.8)	8.2 (8.1, 8.3)
Marathon	5.3 (5.2, 5.4)	5.9 (5.9, 6.0)	6.7 (6.7, 6.8)	7.7 (7.6, 7.8)	8.2 (8.1, 8.3)
Pentathlon	5.4 (5.4, 5.5)	5.9 (5.9, 6.0)	6.8 (6.8, 6.9)	7.7 (7.6, 7.8)	8.2 (8.1, 8.3)
Rowing	5.4 (5.4, 5.5)	5.9 (5.9, 6.0)	6.9 (6.8, 7.0)	7.8 (7.7, 7.9)	8.3 (8.1, 8.4)
Soccer	5.4 (5.3, 5.5)	5.9 (5.8, 5.9)	6.8 (6.8, 6.9)	7.7 (7.6, 7.8)	8.2 (8.1, 8.4)
Swimming	5.4 (5.4, 5.5)	5.9 (5.9, 6.0)	6.8 (6.8, 6.9)	7.7 (7.7, 7.8)	8.2 (8.1, 8.4)
Tennis	5.3 (5.4, 5.5)	5.9 (5.9, 6.0)	6.8 (6.8, 6.9)	7.7 (7.6, 7.8)	8.2 (8.1, 8.4)
Triathlon	5.4 (5.3, 5.5)	5.9 (5.9, 6.0)	6.8 (6.8, 6.9)	7.8 (7.7, 7.9)	8.3 (8.1, 8.4)
Volleyball	5.4 (5.4, 5.6)	5.9 (5.9, 6.0)	6.8 (6.7, 6.9)	7.7 (7.6, 7.8)	8.2 (8.1, 8.3)
Waterpolo	5.4 (5.4, 5.6)	5.9 (5.8, 5.9)	6.8 (6.8, 6.9)	7.8 (7.7, 7.9)	8.3 (8.2, 8.4)
Sport modality					
Endurance	5.3 (5.2, 5.4)	5.9 (5.8, 5.9)	6.8 (6.7, 6.9)	7.7 (7.7, 7.8)	8.2 (8.2, 8.3)
Velocity/power	5.5 (5.4, 5.6)	6.0 (5.9, 6.1)	6.8 (6.6, 6.7)	7.7 (7.6, 7.7)	8.2 (8.1, 8.3)
Team sports	5.4 (5.3, 5.5)	6.0 (5.9, 6.1)	6.8 (6.8, 6.9)	7.7 (7.6, 7.8)	8.3 (8.2, 8.3)

## Data Availability

Data can be obtained from Francesco Campa on francesco.campa3@unibo.it.
